# Air artifact suppression in phase contrast micro-CT using conditional generative adversarial networks

**DOI:** 10.1107/S1600577525001511

**Published:** 2025-03-26

**Authors:** Md Motiur Rahman Sagar, Lorenzo D’Amico, Elena Longo, Irma Mahmutovic Persson, Richard Deyhle, Giuliana Tromba, Sam Bayat, Frauke Alves, Christian Dullin

**Affiliations:** a Translational Molecular Imaging, Max-Plank-Institute for Multidisciplinary Sciences, Germany; b Elettra-Sincrotrone Trieste SCpA Italy; c Department of Physics University of Trieste Italy; d Medical Radiation Physics, Department of Translational Medicine Lund University Sweden; e Inserm UA07 STROBE Laboratory Université Grenoble Alpes France; f Clinic for Haematology and Medical Oncology University Medical Center Göttingen Germany; g Institute for Diagnostic and Interventional Radiology University Medical Center Göttingen Germany; h Diagnostic and Interventional Radiology University Hospital Heidelberg Germany; i Translational Lung Research Center Heidelberg German Center for Lung Research Germany; Tohoku University, Japan

**Keywords:** air artifact suppression, phase contrast micro-CT, FFPE lung tissue, virtual histology, conditional GANs

## Abstract

3D virtual histology of formalin-fixed and paraffin-embedded (FFPE) tissue by means of phase contrast micro-computed tomography (micro-CT) is an increasingly popular technique, as it allows the 3D architecture of the tissue to be addressed without the need of additional heavy ion based staining approaches. Therefore, it can be applied on archived standard FFPE tissue blocks. However, one of the major concerns of using phase contrast micro-CT in combination with FFPE tissue blocks is the trapped air within the tissue. While air inclusion within the FFPE tissue block does not strongly impact the workflow and quality of classical histology, it creates serious obstacles in 3D visualization of detailed morphology. In particular, the 3D analysis of structural features is challenging, due to a strong edge effect caused by the phase shift at the air-tissue/paraffin interface. Despite certain improvements in sample preparation to eliminate air inclusion, such as the use of negative pressure, it is not always possible to remove all trapped air, for example in soft tissues such as lung. Here, we present a novel workflow based on conditional generative adversarial networks (cGANs) to effectively replace these air artifact regions with generated tissue, which are influenced by the surrounding content. Our results show that this approach not only improves the visualization of the lung tissue but also eases the use of structural analysis on the air artifact-suppressed phase contrast micro-CT scans. In addition, we demonstrate the transferability of the generative model to FFPE specimens of porcine lung tissue.

## Abstract 2

A novel workflow using conditional generative adversarial networks (cGANs) to remove/suppress air artifacts in phase contrast micro-computed tomography, improving visualization and facilitating structural analysis, is described.

## 1. Introduction

Evaluation of biomedical tissue at the cellular level is crucial for medical diagnosis as well as for fundamental research. For this purpose, typically histology – the analysis of micrometre thin stained tissue sections using optical microscopy – is commonly applied. Histology requires mechanical sectioning of the specimens, which is facilitated by embedding them in a stiff matrix, most often in paraffin. Despite the great success of histology, its intrinsic 2D nature hinders the evaluation of 3D tissue structures such as fiber orientation, position of meta­stasis or tissue architecture in general. Nowadays, micro-computed tomography (micro-CT) is increasingly used for 3D tissue analysis (Albers *et al.*, 2018 ▸; Metscher, 2021 ▸; Wells *et al.*, 2021 ▸). However, due to limited soft-tissue contrast in classical micro-CT, such an approach often requires additional staining with heavy elements (Metscher, 2009 ▸), which in turn renders sample preparation more complex. Phase contrast micro-CT (PCµCT) is a technique in which the contrast is more related to the scattering and refraction of the incident X-ray beam (Momose *et al.*, 1996 ▸) than to its attenuation like in classical computed tomography (CT). PCµCT strongly increases soft-tissue contrast without additional staining, and thus has been recently used in various studies (Kitchen *et al.*, 2017 ▸; Saccomano *et al.*, 2018 ▸; Töpperwien *et al.*, 2018 ▸; Donato *et al.*, 2024 ▸; Walsh *et al.*, 2021 ▸).

**Figure 1:**
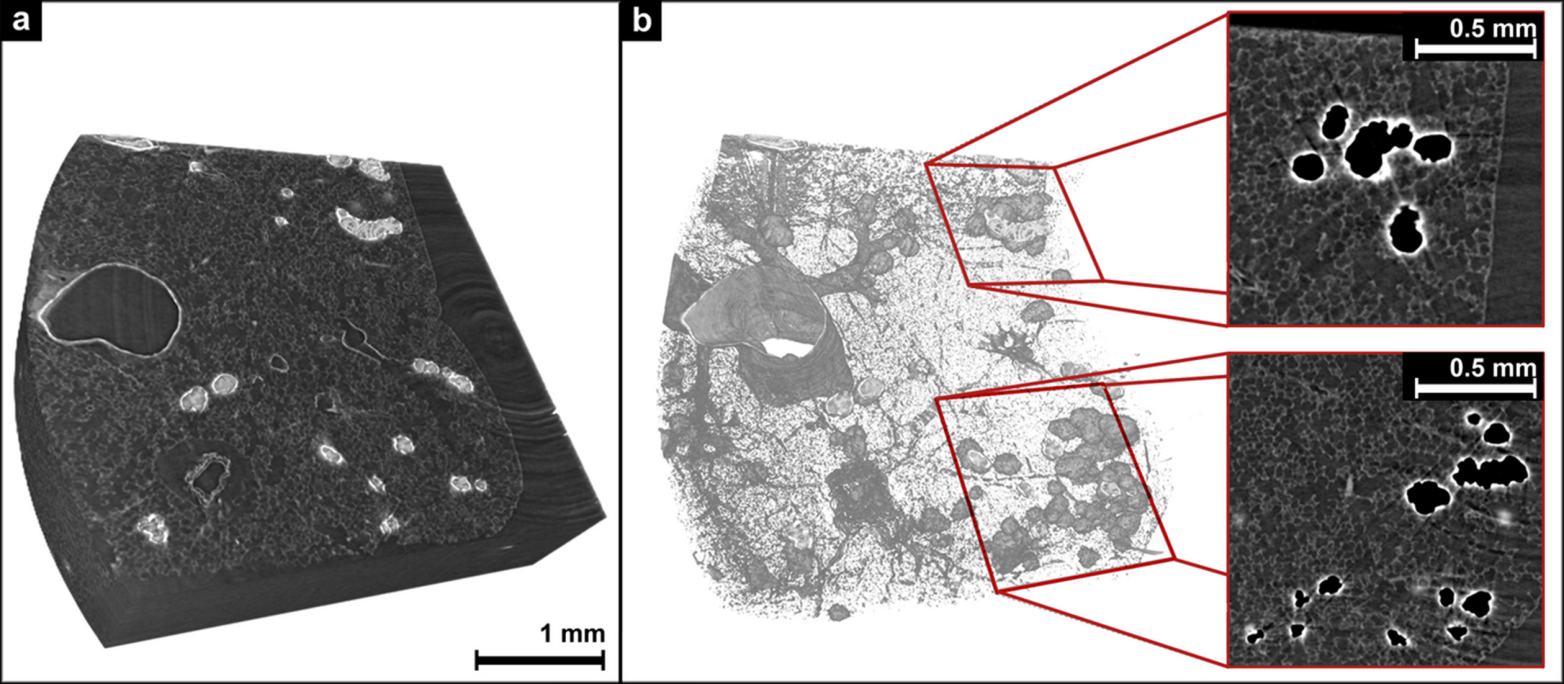
Visualization of air artifacts in a PCCT scan of a FFPE tissue block of a rat lung. (*a*) A 3D chunk of FFPE lung tissue where artifacts due to trapped air bubbles are clearly visible. (*b*) The same chunk as in (*a*) with a threshold higher than the intensity of paraffin. The visualization reveals the 3D structure of the air artifacts and illustrates the 2D view of the air artifacts with the two marked regions (red rectangle).

Among many other methods, formalin-fixed and paraffin-embedded (FFPE) tissue is by far the most utilized tissue preserving protocol for classical histology as well as subsequent analysis. Although FFPE is well suited for both classical and virtual histology using micro-CT, one of the main challenges of using it in combination with PCµCT is the inclusion of air bubbles on the surface or within the tissue. Typically, entrapped air bubbles within the FFPE blocks do not affect the quality of the histology. However, they can have a major impact on the visualization and analysis (Donato *et al.*, 2024 ▸; Strotton *et al.*, 2018 ▸; Norvik *et al.*, 2020 ▸; Handschuh *et al.*, 2022 ▸) of PCµCT scanned tissue samples. Visualization as well as structural tissue analysis becomes extremely difficult due to the high contrast caused by the phase shift at the air-tissue/paraffin interface. Approaches such as formalin fixation and tissue embedding in combination with negative pressure can effectively remove the majority of air bubbles at the tissue surface (Brunet *et al.*, 2023 ▸). However, it is not always possible to remove all air bubbles, especially for lung tissue where air is trapped inside small structures such as the alveoli. Fig. 1 ▸ shows part of a PCµCT scan from an FFPE tissue block of a rat lung with air artifacts. 3D rendering reveals air bubbles on the surface [Fig. 1 ▸(*a*)] as well as within the lung tissue [Fig. 1 ▸(*b*)]. The 2D slices in the marked region (red rectangles) illustrated in Fig. 1 ▸(*b*) reveal voids surrounded by bright bands caused by the air artifacts. These air artifacts arise from the use of a single-distance phase retrieval algorithm, where only the interface between two specific materials – lung tissue and paraffin – remains well defined, while other interfaces, such as air-to-paraffin or air-to-lung, retain strong phase contrast edge-artifacts. Even the stepwise phase retrieval approach for different interfaces, as proposed by Beltran *et al.* (2011 ▸), cannot be applied effectively, as the interfaces are not sufficiently spatially separated. As a result, air, particularly in the peripheral airways, causes strong artifacts that merge the airways, complicating their morphological analysis. However, unlike metal artifacts in classical CT, air artifacts in PCµCT affect a much smaller proximity and do not interfere with the quantification of more distant structures.

Quantifying 3D tissue morphology provides valuable insights into the presence and extent of diseases, as well as the effectiveness of treatments. This is particularly important for lung tissue, where structural integrity is essential for proper respiratory function. In research, such quantification is often carried out by analyzing micro-CT data sets from small animal disease models (De Langhe *et al.*, 2012 ▸). However, due to the lung’s complex hierarchical structure and its small size, accurately segmenting specific generations of the airway tree remains a significant challenge. Structural analysis is typically performed using 3D regions-of-interest (ROIs), which should ideally contain only similar airway generations to avoid confounding effects. These ROIs are usually placed in peripheral lung regions, where airway structures are less heterogeneous. To minimize the influence of artificial cuts at the boundaries of the airways, it is crucial that the selected ROIs are sufficiently large. The placement of these ROIs becomes even more difficult in the presence of air artifacts. Air artifacts, if not properly accounted for, can lead to inaccurate segmentation and hinder the reliability of the analysis. Therefore, careful selection and placement of ROIs is essential to mitigate the impact of both airway complexity and imaging artifacts.

Several studies have used different approaches to address the issue of air artifacts. To exclude air artifacts as features in segmentation and visualization, Lawson *et al.* (2021 ▸) created a binary mask containing void and surrounding bright bands of air bubbles, which was used to replace the corresponding voxels with a fixed value. Katsamenis *et al.* (2019 ▸) proposed the calibration of micro-CT data similar to clinical CT to get rid of air artifacts. Both of the aforementioned methods replace air artifacts with a fixed value, which in turn results in a homogeneous artificial structure within the micro-CT scan. Compensating the artifacts with morphology operations used by Wollatz *et al.* (2017 ▸) is also limited as the used morphology filters need to be adapted to the size of the air artifacts.

Here we present a novel approach utilizing generative adversarial networks (GANs), specifically the conditional variant (cGANs), to replace air artifacts with generated structures that are influenced by the surrounding materials. GANs have been used in many computer vision tasks including medical image analysis. The main idea of GANs is to generate synthetic outputs that appear to be drawn from the distribution of the target domain from which the network has been trained. While GANs can produce outputs by taking random noise as inputs, cGANs take additional information to generate outputs that are precisely controlled. There are many variants of cGANs (Zhu *et al.*, 2017 ▸; Choi *et al.*, 2018 ▸; Liao *et al.*, 2022 ▸), among which pix2pix conditional GANs (pix2pix-cGANs) (Isola *et al.*, 2017 ▸) is the most general supervised image-to-image translation framework. In this framework, the generation of the output image is conditioned on an input image. Our proposed workflow creates source-to-target pairs and trains a network based on pix2pix-cGANs that learns to remove/suppress the air artifacts from PCµCT scans. The efficacy of the trained pix2pix-cGANs model was further evaluated through quantitative and qualitative analysis of the generated data, demonstrating superior rendering for visualization and easing structural analysis in the peripheral lung on the air artifact-suppressed PCµCT scans.

## 2. Materials and methods

### 2.1. Sample preparation

Rat lungs were collected from an approved animal experiment (details in Section 2.5) to study the effect of injurious ventilation in a rat model of ventilator-induced lung injury (VILI). The experiments were performed on 24 Sprague-Dawley rats, with an average weight of 399 ± 26 g. After baseline image acquisition, injurious ventilation was initiated by increasing peak respiratory pressure to 41 ± 2 cm H_2_O, and 0 PEEP (where PEEP refers to the positive end-expiratory pressure), while the respiratory rate was reduced to 30 beats min^−1^ for 20 min to induce VILI. At the end of the *in vivo* imaging, the rats were euthanized by intraperitoneal injection of pentobarbital sodium (Dolethal, 200 mg kg^−1^ body weight; Vetoquinol, Lure, France) and the heart and lungs were excised and removed en bloc for histological analysis. The left lungs were fixed in 4% paraformaldehyde (PFA) at a pressure of 20 cm H_2_O, dehydrated with a graded ethanol series and embedded in paraffin for further histological and post-mortem imaging studies.

Pig lungs were obtained from a licensed slaughterhouse in Germany and kept frozen at −20°C. Lungs were transported to the SYRMEP beamline of the Italian synchrotron ‘Elettra’ in Trieste and slowly defrosted at room temperature approximately 5 h before the phase contrast CT experiment using an athropomorphic human chest phantom (Albers *et al.*, 2023 ▸). The lung was kept within the phantom and fixed using PFA vapor while a constant negative pressure was applied to keep the lung inflated – a technique adapted from Weibel & Vidone (1961 ▸). After approximately 6 h of fixation the lung was removed from the phantom, cut and an approximately 1 cm × 2 cm × 3 cm piece of the right medial lung lobe was extracted, chemically dried using an ascending ethanol series and embedded into paraffin.

### 2.2. Micro-CT acquisition

PCµCT scans of FFPE tissue blocks from rat and pig lung specimens were acquired at the SYRMEP beamline of the Italian synchrotron ‘Elettra’ in Trieste, Italy. The beamline was operated in white beam mode with the following parameters: average photon energy *E* = 23.6 keV and sample-to-detector distance =150 mm, to enable the manifestation of the phase effects. A single-distance phase-retrieval algorithm [homogeneous case of the transport of intensity equation (TIE-HOM) (Paganin *et al.*, 2004 ▸)] was applied with a δ-to-β ratio of 100. This δ-to-β ratio was found to be ideal for the lung tissue to paraffin interface (see Fig. S5 of the supporting information). Finally, a filtered back projection algorithm was employed to reconstruct the PCµCT scans, yielding a 3D dataset with a voxel size of 2 µm × 2 µm × 2 µm (or 4 µm × 4 µm × 4 µm in the case of the pig lung specimen). Detailed parameters can be found in Table 1 ▸. These datasets predominantly expressed the distribution of the phase shift component ‘δ’ of the complex refractive index within the lung.

### 2.3. cGANs

GANs are a type of neural network that consist of a generator and a discriminator network. The fundamental concept of GANs is to generate synthetic outputs that closely resemble the distribution of the target domain. The first introduction of GANs (Goodfellow *et al.*, 2020 ▸) can be referred to as unconditional GANs, in which the generator takes random noise as input and learns to produce convincing synthetic outputs from the real input observations. However, there is no control over the generated output. In the case of cGANs, additional information is provided during the generation process so that GANs can be controlled to generate output according to the condition. This additional information may take the form of a label, text, mask, image or any other attributes to serve as condition to the cGANs. The supervised image-to-image translation model, pix2pix-cGANs, is conditioned on a target image. The generator network in pix2pix-cGANs is a U-Net based on convolutional blocks. Although U-Net was initially developed as a segmentation tool (Ronneberger *et al.*, 2015 ▸), it has proven to be effective for image generation tasks (Hu *et al.*, 2019 ▸; Zeng *et al.*, 2021 ▸; Sorokina & Ablameyko, 2023 ▸) due to its robust encoder–decoder network architecture with skip connections. The discriminator in pix2pix-cGANs, defined as PatchGAN, is a classifier network that is also based on convolutional blocks. PatchGAN takes the output from the generator as input and classifies it as real or fake. The detailed network architecture of the generator and discriminator is described in Section 3.2 below.

#### 2.3.1. Loss function

The loss function of a pix2pix-cGAN is a combination of two components: the adversarial loss and the 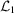 loss. The adversarial loss of a cGAN can be expressed as 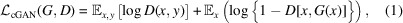
where, with an input *x*, the generator *G* tries to minimize it to produce an output *G*(*x*) that is as close as possible to the ground truth *y*, while the discriminator *D* attempts to maximize it to detect the generated fake output with the greatest precision possible. The adversarial loss is combined with the 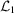 loss (absolute mean error between the ground truth and the generated output) that helps the generator to produce output similar to the corresponding ground truth. 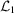 can be written as 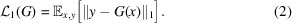
So, the final combined minmax objective function can be written as 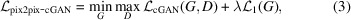
where λ is a hyperparameter that controls the importance of the 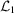 loss relative to the adversarial loss, which is set to 100 as described by Isola *et al.* (2017 ▸).

#### 2.3.2. Model evaluation

In traditional neural networks, the training objective is to minimize the loss function until convergence. The training progression of the networks can be tracked by observing the loss function over training iterations. However, there are no such metrics to assess the training progression and convergence in the case of GANs training. The training of GANs is considered to be converged when both the discriminator and generator losses have reached an equilibrium state. It is not only generally challenging to reach convergence in GANs training but also difficult to measure the training progression solely based on the losses. To obtain a more comprehensive evaluation of the training progression, a generator model was saved after a certain interval (every ten epochs). The saved models were evaluated quantitatively on the test dataset to identify the optimal generator model. The test dataset consisted of 95 pairs of generated and ground truth volumes. To assess how well a model was performing, the relative error (RE) between the generated voxel intensities and the corresponding ground truth voxel intensities was calculated. The RE provides a measure of the difference between the two sets of data, normalized by the intensity of the ground truth values, making it less sensitive to overall scale differences and better suited for comparing models that might generate volumes with different intensity distributions.

The formula for calculating the RE between the generated and ground truth volume pairs is
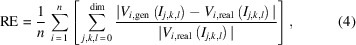
where *n* is the total number of volume pairs in the test dataset (in this case, *n* = 95); *V*_*i*,gen_ and *V*_*i*,real_ are the *i*th generated and corresponding ground truth volume pair, respectively, and*I*_*j*,*k*,*l*_ refers to the intensity of the relevant voxel in the 3D coordinate system (*j*, *k*, *l*), which is the value being compared between the generated and ground truth volume pairs.

A lower RE indicates that the generated volume is closer to the ground truth, meaning that the model is performing better in terms of accurately predicting the voxel intensities. Using RE as a metric is particularly valuable when assessing the effectiveness of generative models in tasks such as denoising, artifact removal or image synthesis, where maintaining the fidelity of the voxel intensities is crucial for the overall quality of the generated output.

The second method employed for the evaluation was the peak signal-to-noise ratio (PSNR). PSNR measures the quality of gray scale generated volume compared with its corresponding ground truth volume pair. PSNR is calculated as 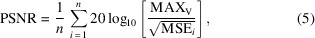
where MAX_V_ is the maximum possible voxel value in the volume (255 for 8-bit gray scale volume), and MSE_*i*_ is the mean squared error between the ground truth and generated *i*th volume pair in the test dataset (*n* = 95). A lower MSE value indicates a similar pair which results in a higher PSNR value. For identical pairs, MSE is zero and PSNR is considered as infinity. PSNR is calculated in decibels (dB) where a higher value suggests better quality.

The third evaluation method used to choose the best performing model is called the structural similarity index measurement (SSIM), which can be expressed as 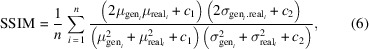
where 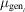 and 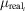 are the mean and 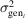 and 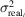 are the variance between the *i*th generated and ground truth volume pair. 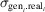 is the covariance between the *i*th generated and ground truth volume pair. *c*_1_ and *c*_2_ are small constants to avoid division by zero. SSIM is a perception-based method that evaluates image quality based on the structural changes while incorporating luminance (brightness) and contrast information. A higher SSIM value (ranging from −1 to 1) indicates a greater similarity between two volumes. In contrast to the RE and PSNR methods, which calculate voxel-wise differences, SSIM assesses the perceived volume quality. The combination of these three methods constituted a comprehensive technique for evaluating the training progression and selecting a generator model for the experiment.

### 2.4. Software

The phase retrieval as well as the reconstruction algorithm on synchrotron data was performed using the software *SYRMEP Tomo Project(STP*) (Brun *et al.*, 2015 ▸). The pix2pix-cGANs model, related functions and all analysis pipelines were implemented in a Python (v3.9) environment. The deep learning framework utilized was *Keras* (Chollet, 2015 ▸) on top of *Tensorflow* (v2.9) (Abadi *et al.*, 2015 ▸). The structural properties of the pores were extracted using an open-source Python toolkit, *PoreSpy* (Gostick *et al.*, 2019 ▸). The pix2pix-cGANs model was trained using a NVIDIA (RTX3090) GPU. The data used in this study can be accessed by a valid request to the correspondence author. The code for the project is available at https://github.com/mrahmansagar/AirGANs.

### 2.5. Ethics

The care of rats and the experimental procedures were in accordance with the Directive 2010/63/EU of the European Parliament on the protection of animals used for scientific purposes and complied with the ARRIVE guidelines (https://arriveguidelines.org/arrive-guidelines). Experimental procedures were evaluated and approved by the local institutional ethical review board and the French Ministry of Higher Education and Research (authorization number: APAFIS#31021-2021040617424365). The animals were housed in a facility with 12 h light/dark cycles, fed *ad libitum*, and were allowed to acclimatize to the housing conditions for a minimum of seven days before any experimental procedures. Pig lungs were obtained from a licensed slaughterhouse, from pigs used for food production. No pig was euthanized for the purpose of this experiment.

## 3. Results

### 3.1. Training data pair preparation

**Figure 2:**
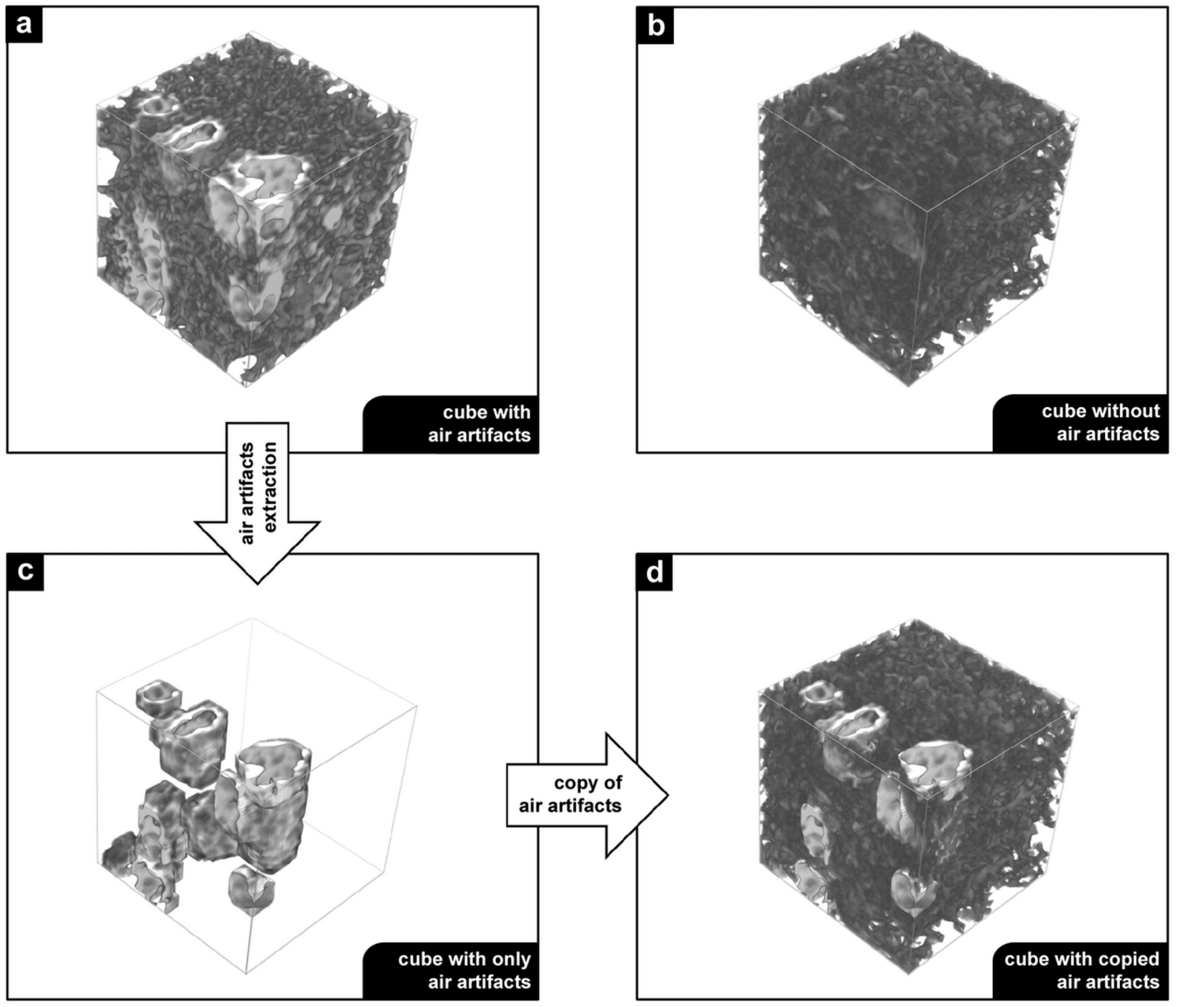
Workflow for creating a data pair to train pix2pix-cGANs. 3D cubes with and without air artifacts are shown in (*a*) and (*b*), respectively. Using a dedicated image processing pipeline, air artifacts are identified and extracted. The extracted air artifacts, illustrated in (*c*), are copied to a cube without air artifacts (*b*). The resulting 3D cube with copied air artifacts is demonstrated in (*d*). This workflow creates source (*d*) and target (*b*) pairs for training the pix2pix-cGANs. The length of each cube is 256 pixels in all directions.

The training of pix2pix-cGANs requires a set of paired source and target domains. In this study, a source and target pair corresponds to a 3D volume with and without air artifacts, respectively. Unlike the applications demonstrated by Isola *et al.* for image-to-image translation (Isola *et al.*, 2017 ▸), manually obtaining a labeled target from a source domain was not feasible in our workflow. Instead, training pairs were created using a semi-automatic workflow. The first step of the workflow was to automatically extract 3D cubes of size 256 × 256 × 256 voxels from the reconstructed PCµCT scans of the FFPE lung tissue blocks. These 3D cubes were then manually classified into two categories: those with and those without air artifacts. Figs. 2 ▸(*a*) and 2(*b*) show examples of a 3D cube with and without air artifacts. Subsequently, the air artifacts were extracted from the cubes with air using a dedicated image processing pipeline. This pipeline comprises two stages: (*a*) creation of a 3D mask of the air artifacts through thresholding, followed by binary morphological operations (opening and dilation) to capture the edges of the air; and (*b*) copying of the masked voxels from the cube with air artifacts to the cube free from air artifacts. An overview of the pipeline for creating a source and target pair is described in the scheme below,[Chem scheme1]
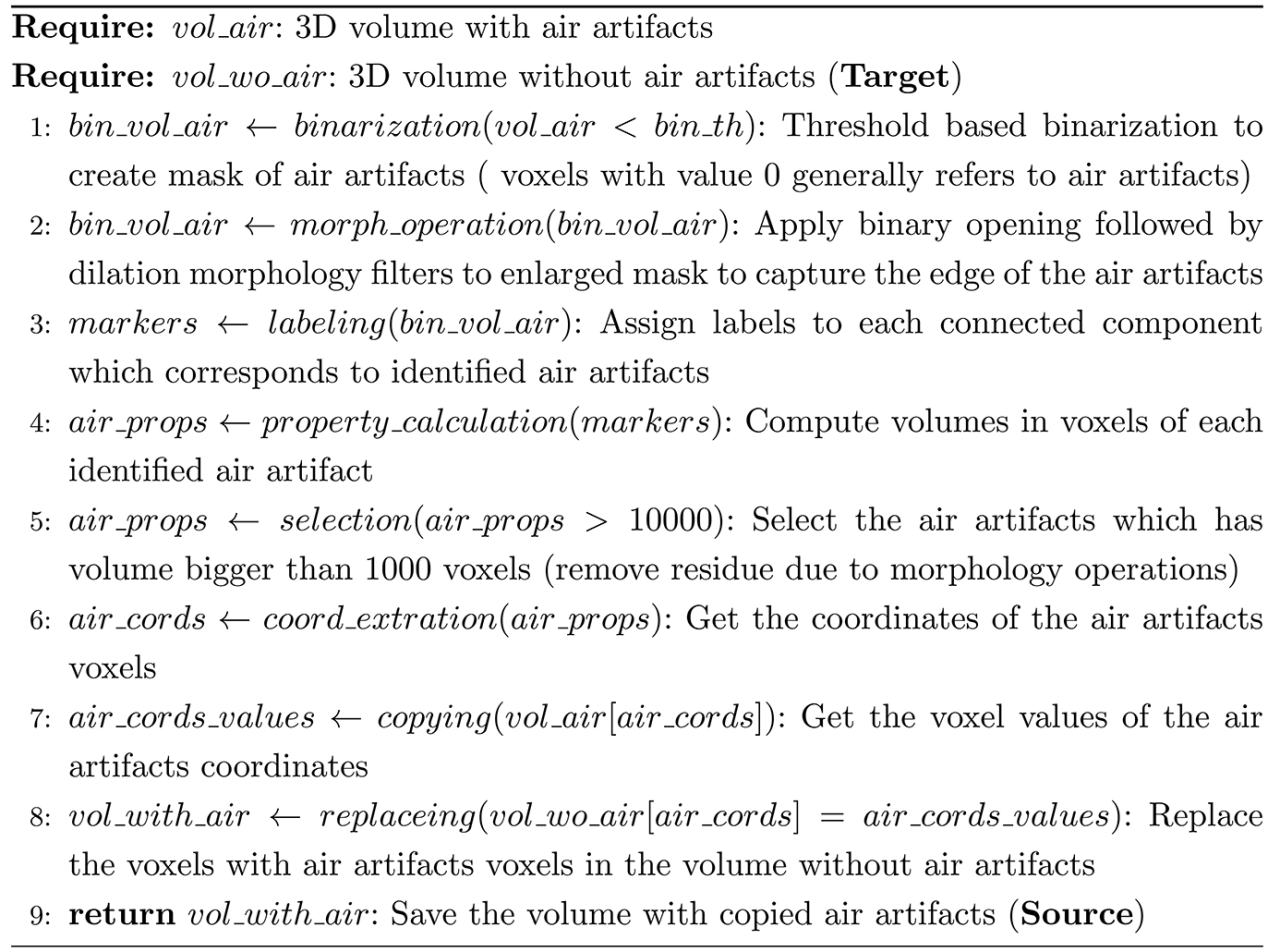
and illustrated in Fig. 2 ▸. Figs. 2 ▸(*b*) and 2 ▸(*d*) represent the target and source cube, respectively. This process was repeated for all the available PCµCT scans, resulting in a training dataset of 900 source and target cube pairs.

### 3.2. Network architecture of pix2pix-cGANs

Pix2pix-cGANs comprise two main parts: a generator and a discriminator network. The generator network is a convolutional-based U-Net that takes an input from the source domain and transforms it into an output of the target domain. The encoder and decoder architecture used for U-Net utilized the same configuration proposed by Isola *et al.* in the original pix2pix-cGANs implementation (Isola *et al.*, 2017 ▸). However, the convolutional blocks were 3D instead of 2D to accommodate the inputs for the study, which were cubes of size 256 × 256 × 256 voxels. The U-Net network architecture is described as below, where C*k* and T*k* refer to convolution and convolution transpose blocks with *k* number of filters, respectively,

Encoder: C64 – C128 – C256 – C512 – C512 – C512 – C512 – C512

Decoder: T512 – T512 – T512 – T512 – T256 – T128 – T64

Each block in the encoder was compiled with a convolution, followed by batch normalization and an activation function, except the first block, where batch normalization was not applied. The configuration of the blocks in the decoder was analogous to that of the encoder, but the convolution was replaced by convolution transpose. Furthermore, a 50% dropout layer was included for the first three blocks. There were skip connections between encoder and decoder blocks at the same resolution level to preserve the important information during the forward pass. The encoder blocks had leaky rectified linear units (ReLUs) with a slope of 0.2 as activation function, whereas the decoder blocks had ReLU activation functions which were not leaky. The last block (bottleneck) of the encoder used a ReLU activation function without any batch normalization layer. To match the output resolution at the end of the decoder, a convolutional transpose layer was utilized with a tanh activation function.

The discriminator network of GANs compares the generator output with the ground truth and classifies it as either real or fake. In the original implementation of the pix2pix-cGANs the discriminator is called a PatchGAN (Isola *et al.*, 2017 ▸). The main idea of the PatchGAN is that, rather than considering the whole input image, it classifies a defined patch across the entire input image and averages the response of each patch to provide the final decision. Each activation output in the receptive field of a PatchGAN represents one area/patch on the input image. The discriminator network architecture can be described as below, wherein C*k* represents a convolution block with k number of filters,

PatchGAN: C64 – C128 – C256 – C512

**Figure 3:**
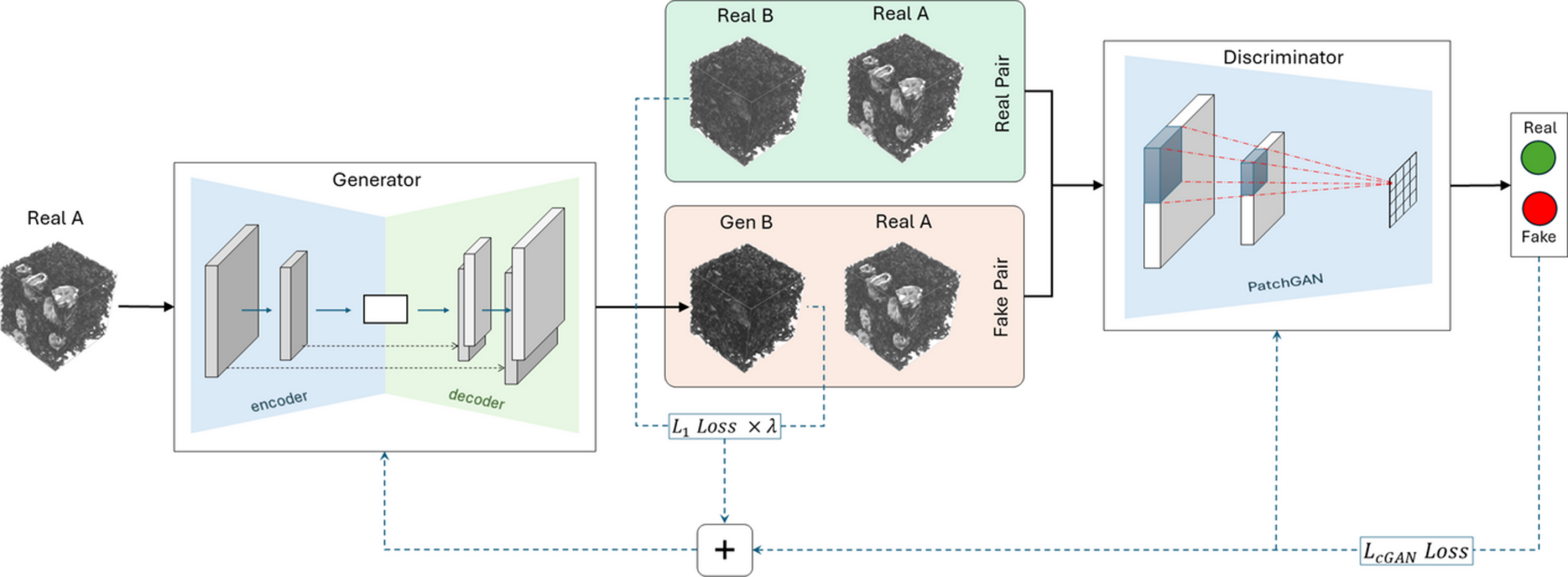
The network architecture of pix2pix-cGANs for translating a 3D cube with air artifacts (source) to a 3D cube without air artifacts (target). The network comprises a generator (U-Net) and a discriminator (PatchGAN), similar to the pix2pix network proposed by Isola *et al.* (2017 ▸), but with network blocks adjusted to accommodate 3D inputs and outputs. The generator receives cubes with air artifacts as input (Real A) and generates cubes which are potentially free from air artifacts as output (Gen B). Pairs of Gen B and Real A (fake pair) are compared with pairs of Real B and Real A (real pair) using the discriminator network. Over the course of prolonged training, the generator network learns to generate outputs that can deceive the discriminator network into misclassifying fake pairs as real ones.

The PatchGAN used in the study shared an identical configuration as implemented in the pix2pix-cGAN (Isola *et al.*, 2017 ▸). However, the convolutional blocks were 3D instead of 2D, resulting in a patch size of 70 × 70 × 70 voxels with a final receptive field of 16 × 16 × 16 voxels. Each convolutional block in the discriminator consists of a convolution operation, followed by batch normalization and a leaky ReLU (slope = 0.2) activation function, with the exception of the first block, in which batch normalization was not employed. To obtain final patch output, a convolution layer was applied with a sigmoid activation function after the last block of the discriminator. The kernel size for all the convolutional operations in the generator and discriminator was set to 4 × 4 × 4 voxels with a stride of 2 × 2 × 2 voxels. This setup enabled downsampling of the input in both the encoder and discriminator while upsampling in the decoder by a factor of two. As an exception, the final two blocks of the discriminator had a stride of 1 × 1 × 1 voxel. Fig. 3 ▸ depicts a schematic of the pix2pix-cGANs network architecture.

### 3.3. Training of the pix2pix-cGANs

The 3D cube pairs (900), prepared with the aforementioned data preparation pipeline (see Section 3.1), were used for training the pix2pix-cGANs. During the training phase, the generator, U-Net, took a cube with air artifacts as input (source: Real A) and was tasked to generate a cube (Gen B) free from air artifacts that closely resembled the ground truth (target: Real B). Both the generated and the ground truth cubes were then combined with the source cube to create a fake and a real pair, respectively. Then, the discriminator, PatchGAN, needed to differentiate between the fake and real pair. Fig. 3 ▸ provides a graphical illustration of the training process. The training process optimized the discriminator’s ability to distinguish real outputs from the generated synthetic outputs. The generator was optimized to minimize the adversarial loss along with the 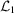 loss, thereby generating outputs that were similar to the ground truth. The generator and the discriminator were trained in an alternative manner, using their respective loss function iteratively with a batch size of 1. The choice of optimizer was Adam with a learning rate of 0.0002 and beta1 = 0, beta2 = 0.999 (Isola *et al.*, 2017 ▸).

### 3.4. Evaluation of generated data

Following the training of the pix2pix-cGANs with the source and target pairs, a generator model was selected based on the criteria mentioned above (see Section 2.3.2). The trained model was then employed to assess the generated air artifact-suppressed cubes of PCµCT scans quantitatively and qualitatively. The assessments were conducted on the cubes (test dataset) that were not included in the training process.

#### 3.4.1. Quantitative assessment

**Figure 4:**
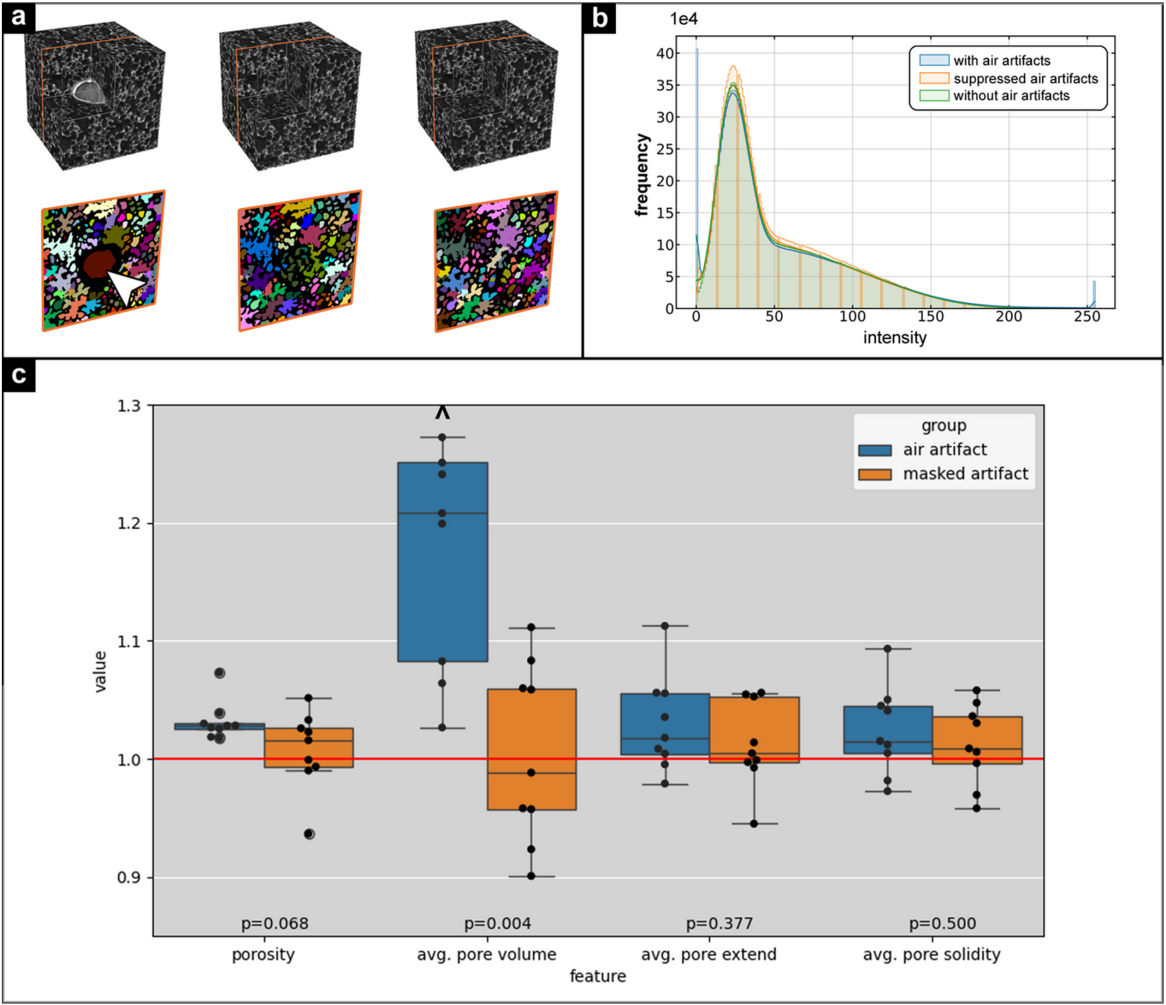
Comparison of generated and actual tissue structures: (*a*) Three 256 × 256 × 256 voxel cubes from a PCCT scan of a rat lung FFPE tissue block: (i) cube with air artifacts (top left), (ii) cube with air artifacts suppressed by a trained generator model (top middle), and (iii) actual cube without air artifacts (top right). (*b*) Histograms of each cube. The cube with air artifacts (blue) shows peaks at 0 and 255, indicating low intensity artifacts and high intensity at their edges. The generated cube (orange) lacks these extremes, aligning with the original cube’s intensity distribution. (*c*) For ten randomly selected ROIs with air artifacts, porosity, pore volume, pore extent and solidity were calculated for the original data (blue) and for data with air artifacts masked (orange). Since there is no ground truth, the artifact-free values (red line, after applying our cGAN) were used as reference. Masking the artifacts resulted in similar measurements to our approach, which increases the average pore size due to blending of alveolar regions (the ^ symbol denotes an outlier).

In order to undertake a quantitative comparison between cubes before and after the suppression of air artifacts, a source to target pair was initially created using the same process as described in Section 3.1. Then the generated cube was compared with the target cube quantitatively. Fig. 4 ▸(*a*) depicts a source (left) to target (right) pair along with generated (middle) cube. One of the quantitative metrics used for comparison was the intensity distribution or the histogram. The histogram of the source (blue), target (green) and generated (yellow) cube are presented in Fig. 4 ▸(*b*). The histogram of the cube with air artifacts exhibited a high frequency at values 0 and 255, which correspond to low intensity voxels within the air artifacts region and high intensity voxels at the edge of the air artifacts, respectively. However, both the generated and target volumes did not display these extreme intensities, which indicates a similarity between the generated and the target volume in intensity distribution.

Additional quantity measures used to assess the structural properties of the generated volume were the extent and the solidity of identified pores of the lung tissue, with extent defined as 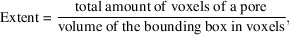
and the solidity as 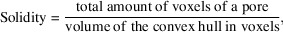
where the convex hull is the the smallest convex polyhedron that encloses all the points (voxels) of the region. A convex hull has no indentations, meaning it is the tightest convex shape that contains the region. A solidity equal to 1 characterizes completely filled convex objects such as a sphere, cube or cylinder. A solidity <1 is found for less compact regions that have gaps, hollow regions or concavities.

The majority of 3D pore segmentation pipelines are effective when the pores are enclosed and have a clearly defined boundary. However, these pipelines are not well suited to the segmentation of 3D pores (airways) of the lung tissue, as the lung tissue is not composed of pores but of strongly connected airways. The 3D airway/pore segmentation pipeline utilized in the study initially segments the pore using a threshold, followed by binary morphology filtering (fill-holes and closing) to eliminate binarization residues. In the next step, a distance transform was applied, resulting in a mask and markers that were essential for the label-based watershed segmentation. The outcome of the watershed segmentation is demonstrated in Fig. 4 ▸(*a*) (bottom row) alongside the corresponding volume (top row). Afterwards, the segmentation, extent and solidity of each identified pore were analyzed. The summary of the pipeline is presented below:[Chem scheme2]
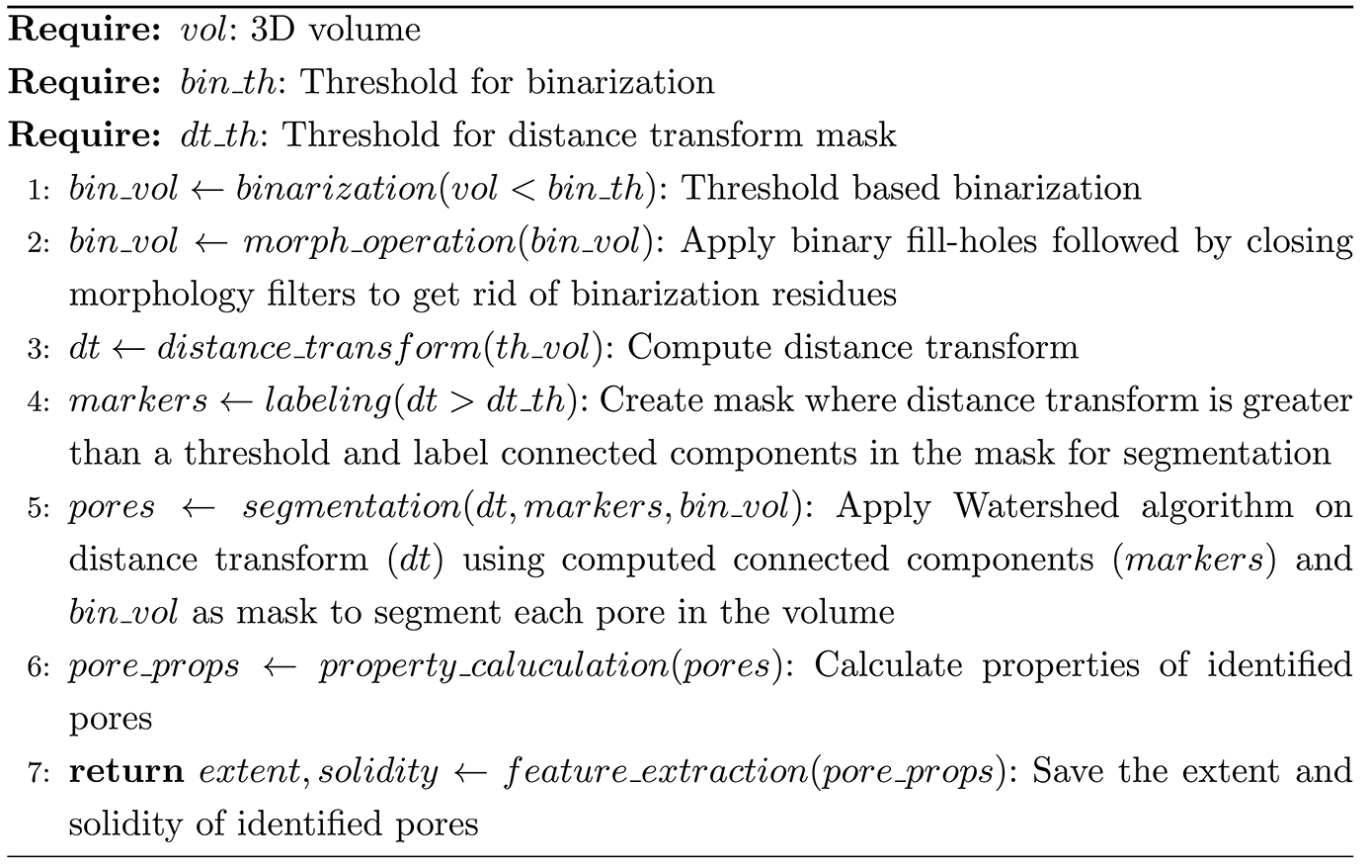


The extent provides insights into the spatial distribution of a pore within a cuboid, whereas solidity offers insights into the density of the pore. These properties together with pore volume and porosity collectively provide a comprehensive structural characterization of the porous medium. Fig. 4 ▸(*c*) presents a comparison of these properties for ten randomly selected cubes with artifacts. Since there is no ground truth, only the quantification with the air artifacts, masking the air artifacts as proposed by Lawson *et al.* (2021 ▸) and after applying our pix2pix-cGANs approach, can be compared. To ease the comparison we used the result of the pix2pix-cGANs for each region and each feature as reverence [Fig. 4 ▸(*c*), red line]. As the air artifacts typically blend multiple alveolar regions together, ignoring them results in strongly increased average pore volume. While masking the artifact region leads to similar results as the pix2pix-cGANs approach, the calculated porosity is on average slightly higher than our approach. Thus, the application of the pix2pix-cGANs method produces similar results as excluding the artifacts from the analysis and at the same time it improves the visualization of those regions, which cannot be achieved by masking the artifact regions.

#### 3.4.2. Qualitative assessment

**Figure 5:**
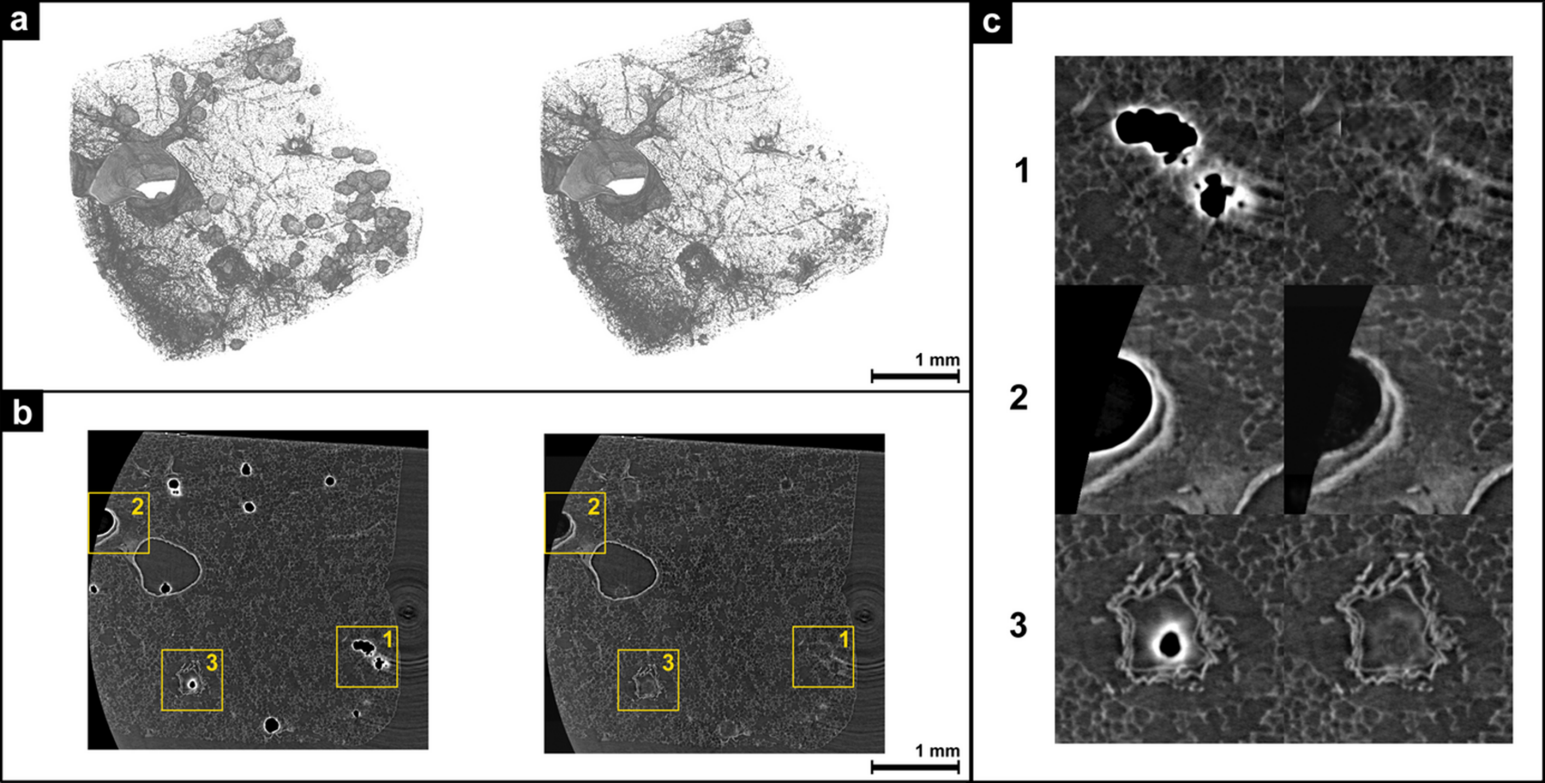
Visual comparison of a PCCT scan of an FFPE rat lung tissue block before and after suppression of air artifacts. (*a*) A 3D rendering of a PCCT scan of FFPE rat lung tissue, with visible air artifacts (left), and the same scan after suppression of these artifacts using a trained pix2pix-cGAN generator model (right). To enhance visualization, a threshold above the paraffin intensity is applied in the 3D rendering. (*b*) A 2D slice from the same scan before (left) and after (right) suppression of air artifacts, corresponding to the dataset shown in (*a*). (*c*) Detailed views [marked in yellow in (*b*)] of specific regions – on the left, the original scan, and on the right, the scan after air artifact suppression. In the first example, air artifacts within the lung parenchyma are replaced with plausible lung tissue structures. In the second, air artifacts in a large vessel are reduced mainly in edge intensity, while, in the third, the artifact in a smaller vessel is replaced with dim, but visibly incorrect, lung tissue. The 3D and 2D visualizations demonstrate that suppression of air artifacts significantly improves the rendering and structural analysis of lung tissue, particularly in the parenchyma regions.

One significant challenge associated with the presence of air artifacts in the PCµCT scans is the difficulty in rendering crucial aspects of the sample for visualization due to the extreme intensity values of these artifacts. For qualitative visual assessment, rendering of the PCµCT scan of a FFPE tissue block was compared before and after the suppression of air artifacts. The visual comparison is demonstrated in Fig. 5 ▸. As the trained model accepts cubes of size 256 × 256 × 256 as input, the initial stage of the process was to identify the cubes containing air artifacts of that size that should be processed by the trained generator of the pix2pix-cGANs model. To achieve this, the entire PCµCT scan was divided into non-overlapping cubes, each with a size of 256 cubic units. Subsequently, each cube was processed to ascertain whether it contained air artifacts. The cubes with air artifacts were then subjected to further processing with the trained generator model. Finally, all the cubes were combined according to their original coordinates to obtain the entire volume of the PCµCT scan. The complete pipeline is summarized in the scheme below:[Chem scheme3]
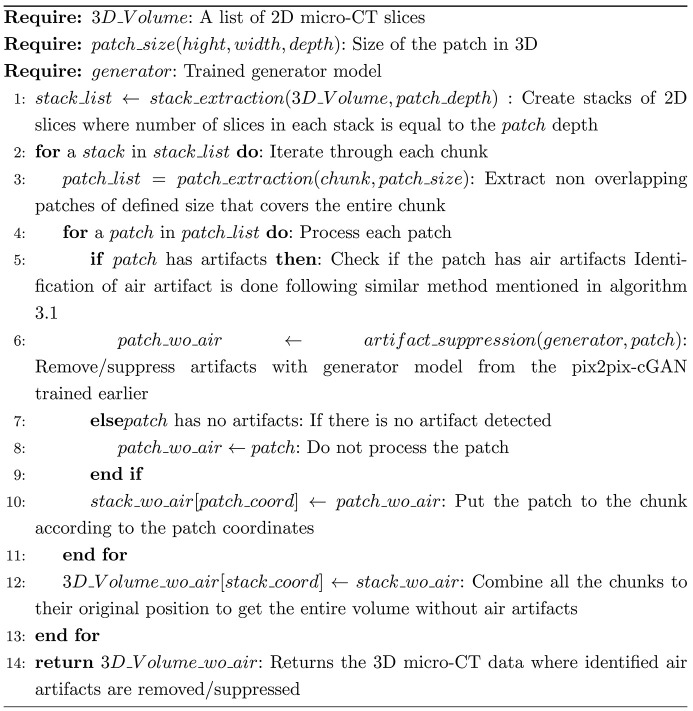


Fig. 5 ▸ demonstrates a visual comparison between a PCµCT scan that contains air artifacts (left) and the same PCµCT scan after the air artifacts were suppressed (right) using the generator from the pix2pix-cGANs model. For better visualization, a threshold value higher than the paraffin intensity was used for the 3D rendering [Fig. 5 ▸(*a*)]. One slice from the corresponding volume above also compares the results in 2D [Fig. 5 ▸(*b*)], showing a notable reduction in air artifacts within the PCµCT scan following suppression. The pix2pix-cGANs model was primarily trained to replace air artifacts in peripheral lung regions. The indicated regions [Fig. 5 ▸(*b*), yellow ROIs] represent an alveolar region (1), a large bronchi (2) and a larger vessel (3). In Fig. 5 ▸(*c*), close-ups of these three regions are shown before (left) and after artifact removal (right). In the first case, the artificially inpainted alveolar structure appears natural, and the artifact is nearly completely removed. In the second case, the strong edge enhancement of the air regions is reduced without introducing artificial tissue structure. In the last case, the artifact was removed, but dim artificial lung tissue was incorrectly inpainted.

## 4. Discussion

Here we demonstrate that artifacts resulting from air inclusion in the PCµCT scans of unstained FFPE lung tissue blocks of a rat VILI model as well as of a porcine lung tissue can be effectively replaced with artificial tissue content through the application of pix2pix-cGANs models. The generated PCµCT scans were free from the extreme contrast caused by air in the specimen, thereby enabling the rendering of the specimen’s overall appearance in 3D without the strong impact of the air artifacts. Furthermore, we showed that, despite the fact that the regions were filled with artificially generated data, pore analysis of the region revealed structural characteristics comparable with those in which the air artifact regions were simply masked, as proposed by Lawson *et al.* (2021 ▸).

Virtual histology of FFPE tissue blocks using PCµCT is an emerging technique (Albers *et al.*, 2018 ▸; Donato *et al.*, 2024 ▸; Peña *et al.*, 2023 ▸; Töpperwien *et al.*, 2016 ▸; Eckermann *et al.*, 2021 ▸) with the primary objective of facilitating a 3D overview of the tissue structure, which can be significantly compromised in the presence of air artifacts. Therefore, the replacement of these artifact regions is of great benefit in this context. Structural analysis of the PCµCT scans with air artifacts would result in the identification of major outliers, which can be avoided if air artifacts are suppressed using the proposed trained generator model of the pix2pix-cGANs.

The air artifacts observed in the PCµCT datasets arise from the use of the single-distance phase retrieval algorithm TIE-hom (Paganin *et al.*, 2004 ▸). This algorithm requires selecting a specific δ-to-β ratio for the material interface of interest, which can result in blurriness or uncompensated edge-enhancement effects at other interfaces. Beltran *et al.* (2011 ▸) proposed an interface-specific improvement to the original TIE-hom algorithm, demonstrating its ability to obtain sharp boundaries at lung-tissue to air and bone to soft-tissue interfaces in *in vivo* PCµCT datasets of young rabbit pups. This method replaces tissue interfaces that retained edge-artifacts from one reconstruction with sharp tissue interfaces from a second reconstruction using a different δ-to-β ratio. However, this approach has a limitation: the tissue interface with retained edge-artifacts is larger than the sharp version, meaning the algorithm can only be applied when the interfaces of interest are sufficiently spatially separated. In our case, the air artifacts are in direct contact with the lung tissue, making it impossible to apply Beltran *et al.*’s method without compromising the surrounding lung tissue. Therefore, we argue that our approach, utilizing a cGANs, is more suitable for reducing these artifacts, especially in this scenario.

The 3D nature of the PCµCT scans enables quantitative analysis of the tissue specimens, as demonstrated in the analysis of lung tissue (Eckermann *et al.*, 2020 ▸; Kampschulte *et al.*, 2013 ▸; Khan *et al.*, 2023 ▸). In general, such analyses are performed on the ROIs which are selected avoiding the air inclusion areas in the specimens. The selection of such ROIs can be a labor-intensive process, often resulting in a few numbers of ROIs per specimen. The outcome of structural analysis conducted with a limited number of ROIs per specimen may be susceptible to selection bias and may lack statistical significance. In this regard, the use of pix2pix-cGANs to suppress/remove air artifacts in the PCµCT scans can facilitate the selection of larger ROI areas that encompass the majority of the specimen, with the positioning of ROI placement being of lesser importance. It is true that the generated structure utilized to fill the artifact region is artificial and lacks pathological significance. Consequently, incorporating it into tissue quantification will result in a deviation from the actual values. However, our data indicate that this discrepancy is negligible in comparison with the substantial impact of having an air inclusion within the measurement region.

The pix2pix-cGANs employed in the study had the identical network architecture proposed by Isola *et al.* (2017 ▸); however, the network blocks were 3D instead of 2D to accommodate the input cube pairs. The training data were prepared with a dedicated data preparation pipeline, exclusively from the PCµCT scans of FFPE tissue blocks of rat lung specimens. Following the training of the 3D pix2pix-cGANs, a generator model was selected to suppress/remove air artifacts in the PCµCT scans of a rat lung, as well as pig lung specimens, although the generator model demonstrated a superior performance on the rat lung data with which it was trained. It also exhibited encouraging outcomes on the pig lung data (see Fig. S4 of the supporting information), which further substantiates the model’s versatility and applicability to diverse PCµCT scans of FFPE lung tissue. To facilitate the training process of the pix2pix-cGANs, pairs of images with and without air artifacts were required. Since it is not possible to acquire the same specimen in the exact position both with and without air artifacts, the training data were generated by simulating these artifacts on otherwise artifact-free regions. Two approaches were considered for this task: (i) artificially generating air artifacts within the lung structure, and (ii) copying existing air artifacts to different locations within an otherwise artifact-free region of the dataset. In the first case, we were unable to generate artifacts with a realistic structure and shape, so we favored the second approach. However, when copying existing air artifacts to other lung regions, it could not be ensured that the anatomical region being replaced was identical to the original one that caused the air artifacts. This limitation reduces the ability of the trained network to effectively replace air artifacts with lung tissue.

Our study has considered exclusively lung tissue specimens, which are particularly susceptible to air artifacts due to the inherent difficulty in completely eliminating air during the sample preparation. However, the novel concept and workflow presented here have the potential to be applied to a diverse range of specimens, offering a promising solution to the challenges posed by artifacts in scientific investigations.

## Figures and Tables

**Table 1:** PCµCT scan parameters

Property	Rat lung tissue	Pig lung tissue
Scan mode	360 off-center	180
Projections	1800	
Exposure time	20 ms	
Pixel size	2 m	4 m
Field-of-view per step	8 mm × 8 mm × 4 mm	8 mm × 8 mm × 8 mm
Sample orientation	Vertical	Vertical
Mosaic scan setup (*X*, *Y*, *Z*)	1, 1, 4 × 3.5 mm	3 × 4 mm, 1, 3 × 5.3 mm
Sample-to-detector distance	150 mm	150 mm
